# The Atlanta Medical Society

**Published:** 1882-09

**Authors:** 


### Societies.

### THE ATLANTA MEDICAL SOCIETY.

*Reported for the Atlanta Medical Register.*

ATLANTA, August, 1882.

Dr. V. H. Taliaferro in the chair.

Dr. G. G. Roy reported a case of fracture of the spinous processes of the second, third and fourth cervical vertebræ, as follow: J. W. W., a white man, aged thirty five years, jumping from a street car lost his balance and sustained a fall, striking the back of his neck against the step of the car, inflicting a severe but obscure injury of the cervical spine and head, besides fracturing the sixth and seventh ribs of his left side. The latter injury caused an effusion into the left pleural cavity, and, penetrating the lungs, resulted in emphysema.

The patient was not rendered unconscious by the fall, neither was there any paralysis, though numbness existed in the muscles of the left side of chest.

The patient was able to walk several squares after receiving the injury, but was unable to move his head in any direction whatsoever, and there was severe pain in the region of the occipital protuberance, which at times was so great as to almost result in convulsions.

He complained of great soreness and stiffness of the muscles of the neck and back.

There was great despondency and nervousness, and in fact all the phenomena that generally follow a case of delirium tremens, were present. He continued in this state until the morning of June 11th, about three months after the receipt of the injury, when after wandering around the neighborhood as usual, for several hours, he went to his room, threw himself on the bed and died immediately.

The post mortem revealed the following lesions:

The spinous processes of the second, third and fourth cervical vertebræ were completely severed from the bodies of the bones and were dislocated to the right. There was marked softening of the brain substance, and a good deal of hemorrhage through the meninges covering the cerebrum, which were perforated. This was due to the inflammation and degeneration of the brain substance. Hemorrhagic effusion also existed between the dura mater and the skull, which was, in the opinion of Dr. R., the immediate cause of death.

The treatment was merely palliative; opium being given to relieve pain, digitalis to regulate the heart's action and veratrum viride to control the pulse. Dr. W. F. Westmoreland and Dr. W. T. Goldsmith visited the case with Dr. Roy.

The cervical portion of the spine—removed at the autopsy—was presented for inspection, plainly showing the lesion indicated in the report.

This report was discussed by Drs. Taliaferro, O'Brien, and Elkin, who expressed the concurrent opinion that the injury caused congestion of the spinal cord, resulting in an inflammation extending to the brain. This caused degeneration of the cerebral substance and of the walls of the blood vessels resulting in hemorrhage and death.

Dr. R. S. Carter reported two cases of syphilis presenting unusual and irregular symptoms. The most prominent being alopecia occurring at a very early stage, and, if the statements of the patients are to be credited, showing a remarkably short period of incubation.

Dr. F. H. O'Brien thought from the brief histories recited, that the infection was the result of former exposures, and that the disease was not contracted at the late period indicated by the subjects of Dr. C.'s report.

Dr. G. G. Roy expressed the opinion that chancre may occur earlier than is generally supposed—ealier than accepted authorities allow. He referred briefly to the history of a young man who suffered from syphilitic alopecia. After successful treatment the new hair came out white.

Dr. W. S. Elkin reported a case of convulsions following delirium tremens. A young man, aged twenty years, who had not drank for months, came to him after a hard days drinking, in great fear of an attack of delirium tremens, having suffered in that way before. While in his office he had a severe convulsion. He administered bromide of potassium, morphine and chloral which brought prompt relief. The next day the man drank again and the convulsions recurred. The same treatment again relieved him. Later in the day he complained of great pain in the throat and stomach and suddenly went into a series of violent convulsions. His throat, during the continuance of the attack, presented the appearance as if the trachea was spasmodically closed, preventing expiration. Within two weeks from that time the man had another similar, but even more severe, attack. The whisky which he took did not make him drunk.

Dr. Roy mentioned the case of a young lady—a teacher in a female seminary—very delicate and a dyspeptic. She ate a hearty dinner, and during the afternoon laid down, complaining of vertigo. She was soon found in a convulsion, which recurred at intervals during the following night. Gave chloroform by inhalation and anti-spasmodics. The abdomen was very tympanitic and greatly distended. Ordered an active cathartic, and after free evacuation of the bowels the convulsions ceased. Thinks she had in all twelve severe and distinct convulsions.

She had fever next day, with occasional tendency to a recurrence of convulsions—which, however, did not fully develop; complained greatly of an uncomfortable feeling in her throat.

Dr. O'Brien considered these hysterical convulsions. The globus hystericus was well marked.

Dr. O'Brien, continuing, reported the case of a burn in the person of a girl aged five years. The child was playing with a match, which ignited and set the sleeve of her dress on fire. The whole arm was burned so that the cuticle came off from the elbow to the shoulder. He applied first lint soaked in a solution of chlorinated soda (Labarraque's solution)—one ounce to a pint of water. After twenty-four hours used carbolized vaseline. The case was marked by very prompt recovery and remarkably slight constitutional disturbance.

Dr. G. H. Noble gave the details of a case, witnessed last winter in a New York hospital, as follows: A woman, aged forty-five years, in good health, after moderate physical exertion, was seized with pain in the lower part of the abdomen, circulation depressed, surface cold. Diagnosis internal hemorrhage—hematocele suspected. A consultation resulted in a disagreement as to diagnosis. She died in a few hours, and the post-mortem revealed a ruptured bladder.

Dr. W. S. Elkin reported the case of a negro woman aged fifty-five years, suffering from diabetis mellitus of several months duration. Thirst excessive and appetite voracious. Passed two and a half gallons of urine in twenty-four hours of a specific gravity of 1018. Fehling's test revealed the presence of sugar. Treatment consisted in regulating the diet, ergot and an iron tonic. The improvement was prompt and has been progressive.

Dr. Roy has used ergot with advantage in these cases, and while on this subject, remarked that the urine of patients is not examined often enough. Its examination frequently discloses disorders that otherwise would prove very obscure, or not be discovered at all.

Recently he tested the urine of an applicant for life insurance, as is required by the rules of some companies. The subject appeared healthy, and entertained no suspicion of any disease, but when the test was applied, two-thirds of the specimen in the tube was found to be albumen. This may appear incredible, but the fact forcibly illustrates the point which he desired to make.

Dr. Elkin agreed with Dr. R., and added that the microscope was also a too much neglected instrument of accurate diagnosis. He referred incidentally to an old man who suffered from a very obscure disease. Several prominent practitioners failed to agree on the diagnosis. Finally the microscope revealed cancer cells in the urine and determined the diagnosis in favor of cancer of the bladder. As the disease soon terminated fatally the autopsy confirmed the correctness of this conclusion.

